# The 26S Proteasome Is Required for the Maintenance of Root Apical Meristem by Modulating Auxin and Cytokinin Responses Under High-Boron Stress

**DOI:** 10.3389/fpls.2019.00590

**Published:** 2019-05-14

**Authors:** Takuya Sakamoto, Naoyuki Sotta, Takamasa Suzuki, Toru Fujiwara, Sachihiro Matsunaga

**Affiliations:** ^1^Department of Applied Biological Science, Faculty of Science and Technology, Tokyo University of Science, Noda, Japan; ^2^Department of Applied Biological Chemistry, Graduate School of Agricultural and Life Sciences, The University of Tokyo, Bunkyō, Japan; ^3^College of Bioscience and Biotechnology, Chubu University, Kasugai, Japan

**Keywords:** auxin, boron, cytokinin, root apical meristem, 26S proteasome

## Abstract

Boron (B), an essential micronutrient, causes adverse effects on the growth and development of plants when highly accumulated. By the analysis of *Arabidopsis* mutants hypersensitive to high-boron (high-B) stress, we have shown that 26S proteasome (26SP) is required to maintain the morphology of the root apical meristem (RAM) under high-B stress. To further understand the molecular function of 26SP in tolerance to high-B stress in the RAM, in this study we investigated the pathways regulated by 26SP using a 26SP subunit mutant, *rpt5a*, which is hypersensitive to high-B stress. Expression of *RPT5a* was induced by high-B stress in the entire RAM accompanied by its strong expression in the stele, including the stem cells. Analysis of stele organization in the *rpt5a* mutant revealed that 26SP is especially important for maintenance of the stele under high-B stress condition (3 mM B treatment). Expression analyses of an auxin-response reporter revealed that auxin responses were enhanced in the stele and the stem cell niche by high-B stress, especially in the *rpt5a* mutant. In contrast, the expression of *TCS::GFP* representing cytokinin signaling in the stem cell niche was unchanged in the wild type and extremely weak in the *rpt5a* mutant, irrespective of B condition. The drastically aberrant auxin and cytokinin responses in the *rpt5a* mutant under high-B stress were supported by transcriptome analysis using root tips. These results suggest that the collapse of hormonal crosstalk in the stele including the stem cells occurred in the *rpt5a* mutant, especially under high-B stress. Treatment with the auxin signaling inhibitor α-(phenyl ethyl-2-one)-indole-3-acetic acid (PEO-IAA) reduced sensitivity to high-B stress in the wild type and restored the RAM morphology in the *rpt5a* mutant under the high-B stress condition. In addition, cytokinin treatment conferred the *rpt5a* mutant with tolerance to high-B stress in RAM morphology. It is concluded that 26SP containing RPT5a is required for maintenance of auxin/cytokinin balance in the stele, which is crucial for preventing defects in RAM morphology under high-B stress.

## Introduction

The micronutrient boron (B) performs indispensable roles for plant growth and development. However, B is toxic when it is accumulated to a high concentration in plant tissues, and consequently causes growth retardation. Given that the optimal range in B concentration in the soil is considered to be narrow, a limitation on crop productivity is often observed, especially in arid and semi-arid areas, including South Australia, Turkey, Mediterranean countries, California, and Chile in addition to regions with high-B soils of anthropogenic origin caused by agricultural irrigation and over-fertilization (Nable et al., 1997; Kot, 2007). Therefore, it is important to understand the mechanism of plant tolerance of high-B stress at the molecular level, which will potentially aid in breeding crops tolerant of B toxicity.

The root tip is the primary belowground target of B toxicity. In wheat roots, application of a high B concentration to mature root tissues other than the root tip has only slight inhibitory effect on root growth (Reid et al., 2004). Two fundamental developmental processes maintain root growth: mitotic cell division in the root apical meristem (RAM), and cell elongation after mitosis (Scheres et al., 2002). Inhibition of cell division in the RAM is a critical cause of B toxicity in roots. In roots of *Arabidopsis thaliana*, high-B treatment decreases the RAM size through inhibition of cell division activity (Sakamoto et al., 2011, 2018; Aquea et al., 2012). The reduction in cell division activity is also observed in root tips of *Vicia faba* treated with a high B concentration (Liu et al., 2000), The inhibition of cell division is attributed to DNA damage caused by high-B stress in the RAM (Sakamoto et al., 2011). We previously reported that a chromosomal protein complex (condensin II) and a regulatory particle AAA-ATPase 5a (RPT5a) of 26S proteasome (26SP) are essential for amelioration of high-B-dependent DNA damage and the maintenance of RAM size in *A. thaliana* (Sakamoto et al., 2011, 2018).

The 26SP protein complex is composed of the 20S core particle (CP) and 19S regulatory particle (RP). The complex regulates numerous biological processes, such as cell-cycle progression, plant hormonal responses, and signaling in response to abiotic and biotic stimuli through the selective degradation of polyubiquitin-tagged proteins (Sadanandom et al., 2012). The *RPT5a* gene belongs to the RP, which functions in the recognition and unfolding of target proteins, and translocation of target proteins to the CP, which shows protease activities (Sadanandom et al., 2012). Loss of function of *RPT5a* gives rise to high-B hypersensitivity that accompanies severe defects in RAM morphology, including disorder of cell alignment around the stem cell niche (Sakamoto et al., 2018). The defects in RAM morphology in the *rpt5a* mutant are partially attributed to failure in degradation of a chromatin remodeling factor, BRAHMA (BRM) (Sakamoto et al., 2018). In the wild type, however, enhanced accumulation of BRM increases sensitivity to high-B stress in root growth without exhibiting the defects in RAM morphology (Sakamoto et al., 2018). This finding implies that RPT5a is also involved in pathways other than the BRM-dependent pathway to maintain RAM morphology under high-B stress.

Maintenance of the stem cell niche is the basis for formation of proper RAM morphology, which is controlled by several hormonal pathways (Lee et al., 2013). Auxin and cytokinin are the principal regulators of RAM maintenance (Moubayidin et al., 2009). Auxin and cytokinin have an essentially antagonistic functional relationship; cytokinin stimulates cell differentiation by suppression of auxin signaling and transport, whereas auxin promotes cell division by inactivation of cytokinin signaling (Lee et al., 2013). Recent studies have revealed crosstalk between auxin and cytokinin signaling in the stem cell niche. The collective activities and topology of the PIN-FORMED (PIN) proteins and the AUXIN RESISTANT 1 (AUX1)/LIKE AUX1 (LAX) family transporter proteins contribute to form the auxin gradient along the RAM and the auxin maximum at the quiescent center (QC) (Blilou et al., 2005; Grieneisen et al., 2007; Ugartechea-Chirino et al., 2010), which produces stem cell initials. This auxin distribution determines the spatial patterning of PLETHORA (PLTs) expression in RAM, which is crucial for the specification of the stem cell niche and to control the proliferation of stem cell daughter cells (Scheres and Krizek, 2018). Auxin-mediated expression of *PLT1* and *PLT2* is regulated by AUXIN RESPONSE FACTOR 5 (ARF5) and ARF7 during the development of the embryonic root (Aida et al., 2004). The ARF5 target TARGET OF MONOPTEROS 5 (TMO5) and its homolog T5L1 promote the periclinal division of procambium cells that function as stem cells of vascular tissues in association with LONESOME HIGHWAY (LHW) in the cytokinin-dependent pathway (De Rybel et al., 2013; Ohashi-Ito et al., 2014).

The ubiquitin/26SP pathway plays critical roles in the regulation of various hormonal signaling pathways (Kelley and Estelle, 2012). The 26SP protein is involved in *ARF* transcription through degradation of the repressor for *ARF* genes, AUX/IAA family proteins ubiquitin-tagged by a E3 ligase complex SCF^TIR/AFB^ in response to auxin perception by TRANSPORT INHIBITOR RESPONSE (TIR1) (Kong et al., 2016). The degradation of AUX/IAA proteins is also mediated by PROTEASOME REGULATOR 1 (PTRE1) dependent on promotion of 26SP activity (Yang et al., 2016). It is suggested that *PLT1* and *PLT2* expression in primary roots is also regulated by BRM action (Yang et al., 2015), whose degradation by 26SP requires RPT5a function (Sakamoto et al., 2018). In addition, the RP subunits RP non-ATPase 10 (RPN10) and RPN12a are involved in the degradation of ARABIDOPSIS RESPONSE REGULATOR 1 (ARR1) and ARR5 (Smalle et al., 2002, 2003; Kurepa et al., 2014). ARR1 is a transcriptional activator that promotes the expression of cytokinin-responsive genes, whereas ARR5 acts in the repression of cytokinin responses (Kurepa et al., 2014), suggesting that 26SP functions in balancing activation and inhibition and/or spatiotemporal regulation of cytokinin responses in roots.

In this study, we characterized the defects in RAM morphology exhibited by the *rpt5a* mutant under high-B stress and showed that 26SP containing RPT5a is involved in adjustment of the activity of TIR1/AFB-dependent auxin signaling to the appropriate level required for the maintenance of the stem cell niche, especially under high-B conditions. In addition, 26SP containing RPT5a is also involved in the regulation of cytokinin responses, which may repress auxin responses and be crucial for stem cell proliferation and consequently the size of the stele in RAM of *A. thaliana*.

## Materials and Methods

### Plant Materials and Growth Condition

The *rpt5a-4* and *rpt5a-6* mutants of *A. thaliana* (background Columbia; Col-0) were established previously (Sakamoto et al., 2018). For reporter analysis, *rpt5a-4* was crossed with reporter lines harboring *pWOX5::erGFP* (Blilou et al., 2005), *DR5::GFP* (Ottenschläger et al., 2003), *pPIN1::PIN1-GFP* (Benková et al., 2003), and *TCS::GFP* (Zürcher et al., 2013). Double-homozygous lines were established from their F_2_ progeny and used for the analysis. In all experiments, seeds were sown on media containing MGRL solution, 1% (w/v) sucrose, and 1.5% (w/v) gellan gum. Boric acid was used to adjust the B concentration in the medium. After 3 days incubation at 4°C, the plates were placed vertically in a growth chamber (16-h light/8-h dark cycle, 22°C) until analysis.

### β-Glucuronidase (GUS) Reporter Line and GUS Staining

The promoter fragment containing 2,735 bp upstream of the start codon of *RPT5a* was amplified from Col-0 genomic DNA using the primers 5′-caccCTCTAGAGGTTCCCAATTAG-3′ and 5′-TCTTCGAAGCTTGACGTATCG-3′, and cloned into the pENTR^TM^/D-TOPO^®^ vector following the manufacturer’s protocol (Invitrogen, Carlsbad, CA, United States). The cloned promoter fragment was subsequently subcloned into pMDC162, a Gateway^TM^ destination vector containing a β-glucuronidase (*GUS*) gene, by LR recombination with LR clonase II (Invitrogen) following the manufacturer’s protocol. The constructs were inserted into *Agrobacterium tumefaciens* (strain GV3101::pMP90) and used to transform Col-0 plants. Transgenic plants were selected on half-strength Murashige and Skoog medium supplemented with 1% sucrose, 20 μg/mL hygromycin B, and 250 μg/mL claforan. Transgenic T_3_ plants harboring homozygous T-DNA were used for subsequent analyses.

To detect GUS activity, seedlings were stained with a solution containing 100 mM Na_2_HPO_4_ (pH 7.0), 0.1% Triton X-100, 2 mM K_3_Fe[CN]_6_, 2 mM K_4_Fe[CN]_6_, and 0.5 mg/mL 5-bromo-4-chloro-3-indolyl-β-D-glucuronic acid for 1 h at 37°C. The GUS-stained seedlings were incubated in clearing solution (80% chloral hydrate and 10% glycerol) overnight at 4°C. Images of GUS-stained roots were captured using a stereomicroscope (SZH10; Olympus, Tokyo, Japan) equipped with a digital CCD camera.

### Root Elongation Assay

Five-day-old seedlings pre-incubated vertically on normal MGRL medium were transferred to fresh medium containing indicated concentrations of B, indole-3-acetic acid (IAA), α-(phenyl ethyl-2-one)-indole-3-acetic acid (PEO-IAA) (Hayashi et al., 2012), and *trans*-zeatin (tZ). Positions of primary root tips were marked on the plates with a pen. After incubation for an additional 4 days, the length of the newly elongated primary roots from the marked positions was determined using ImageJ ver.1.51h software^1^.

### Confocal Fluorescence Microscopy

To observe the RAM structure and expression patterns of reporter genes, roots of 9-day-old seedlings subjected to the 4-day B treatments were stained with propidium iodide (PI) (10 mg/mL; Molecular Probes, Eugene, OR, United States) for 5 min.

For analysis of the number of cell files in the stele, roots of 9-day-old seedlings subjected to the 4-day B treatments were fixed with 4% formaldehyde in PEM buffer [50 mM PIPES (pH 6.8), 2 mM EGTA (pH 7.0), 2 mM MgSO_4_] for at least 24 h at 4°C. The fixed plants were treated with PEMT buffer [1% (v/v) Triton-X 100 in PEM buffer] for 20 min. The nuclei were stained with 1:5000 SYBR^TM^ Green I in PEMT buffer for 10 min. After washing twice with PEM buffer, cell walls were stained with Calcofluor White (Sigma-Aldrich, Tokyo, Japan) for 30 min. After washing twice with PEM buffer, plants were mounted in 20% 2,2′-thiodiethanol in PEM for observation.

The samples were examined using a confocal fluorescence microscope (FV-1200; Olympus). Signals for PI were excited using the 559 nm wavelength and detected using a 575–675 nm band bass filter. Signals for GFP and SYBR Green I were excited using the 473 nm wavelength and detected using a 490–540 nm band bass filter. Signals for Calcofluor White were excited using the 405 nm wavelength and detected using a 430–455 nm band bass filter.

### RNA Sequencing and Data Analysis

The primary root tips (∼1 cm from the tip) were collected from Col-0 and *rpt5a-4* plants treated with 0.03 or 3 mM B. Total RNA was extracted from collected root tips using RNeasy^®^ Plant Mini Kit (Qiagen, Valencia, CA, United States) and treated with RNase-free DNase (Qiagen). A total of 500 ng RNA was subjected to mRNA isolation and subsequent library preparation with the NEBNext^®^ Poly(A) mRNA Magnetic Isolation Module (NEB, Ipswich, MA, United States) and NEBNext^®^ Ultra RNA Library Prep Kit for Illumina (NEB), respectively. The prepared library was sequenced using the NextSeq^TM^ 500 system (Illumina, San Diego, CA, United States) in the single-end mode with a read length of 75 bp. Four independent biological replicates were analyzed for each genotype. The sequencing data are deposited with DDBJ (accession number DRA008073).

The produced bcl files were converted to fastq files by bcl2fastq (Illumina). Quality-filtered reads were mapped onto the Arabidopsis reference genome (TAIR10) by bowtie 1.2.1 (Langmead et al., 2009) with the following parameters: ‘-all -best -strata.’ Up- and down-regulated genes were extracted by R ver.3.2.0 software using the R package edgeR (Robinson et al., 2010), treating biological quadruplicates as paired samples. Genes showing a *p*-value less than 0.05 and more than two-fold change in comparison with the data for Col-0 under the normal B condition (0.03 mM B) were identified. Gene ontology (GO) analysis was performed using GO Enrichment Analysis^2^.

## Results

### RPT5a Is Involved in Maintenance of the Stem Cell Niche Especially Under High-B Stress

**FIGURE 1:**
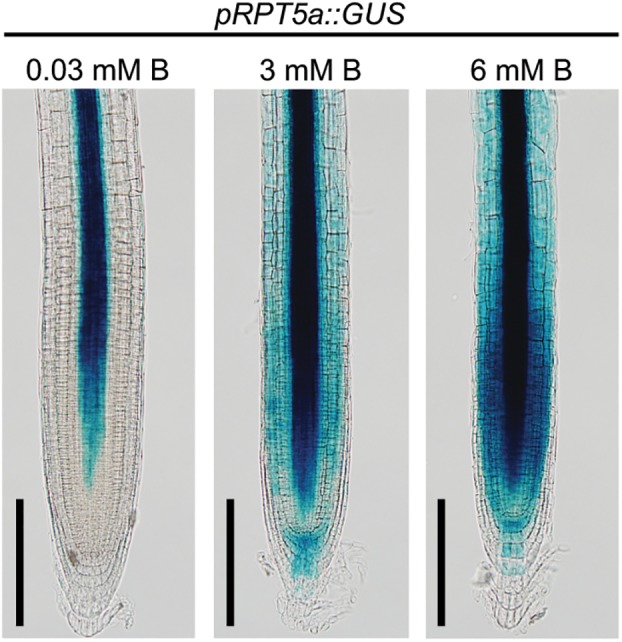
Response of *pRPT5a*::GUS expression to the treatment of high-B stress in the RAM of Col-0. The treatment 0.03 mM B represented the normal B condition; 3 and 6 mM B represented the high-B stress conditions. Scale bars, 100 μm. An additional two independent transgenic lines showed similar responses in GUS expression to the high-B treatments.

Previously, we revealed that RPT5a proteins are expressed in the entire RAM and are essential for maintenance of RAM morphology, particularly under the high-B condition (Sakamoto et al., 2018). To further understand the function of RPT5a in RAM maintenance under high-B stress, we assessed the promoter activity of *RPT5a* using plants with *pRPT5a::GUS* (Figure 1). Under the normal B condition (0.03 mM B), GUS expression was detectable only around the stele, which consists of the primary vascular system, except for the stem cell niche in the RAM. In plants treated with 3 mM B, GUS expression was also detected in the cells outside of the stele in the RAM and staining in the stele extended toward the stem cell niche. Treatment with 6 mM B further enhanced GUS expression in the same regions as observed in the 3 mM B condition. These results indicated that RPT5a in the RAM is responsive to high-B stress.

**FIGURE 2:**
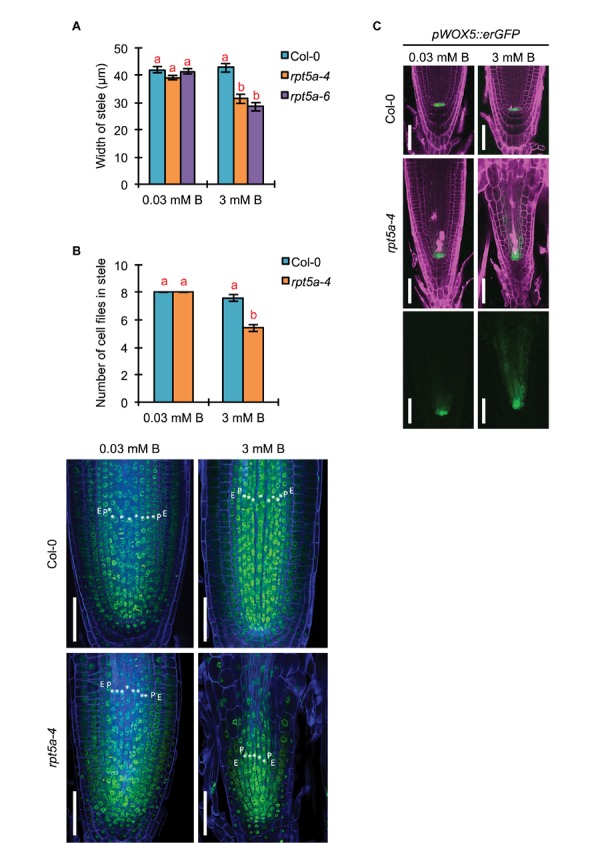
Effects of high-B stress on tissue maintenance in the RAM of the *rpt5a* mutant. **(A)** Width of the stele at the border of the RAM in Col-0 and *rpt5a-4* under the normal (0.03 mM B) and high-B (3 mM B) conditions. Values are means ± SE (*n* = 10, *p* < 0.05, one-way ANOVA and Tukey’s HSD). **(B)** Maximum number of cell files in the stele in Col-0 and *rpt5a-4* under the normal (0.03 mM B) and high-B (3 mM B) conditions. Values are means ± SE (*n* = 5, *p* < 0.05, one-way ANOVA and Tukey’s HSD). Representative images are shown. ^∗^, stele cells; E, endodermis; P, pericycle. Scale bars, 50 μm. Blue, Calcofluor White-stained cell wall. Green, SYBR Green I-stained nuclei. **(C)** Expression patterns of *pWOX5*::*erGFP* in Col-0 and *rpt5a-4* under the normal and high-B conditions. Scale bars, 50 μm. Magenta, PI-stained cell walls; green, GFP fluorescence.

Given that strong promoter activity of *RPT5a* was observed in the stele even under the normal B condition, we investigated the involvement of RPT5a in stele organization by measuring the width of the stele in the RAM. The width of the stele was comparable between the wild type and *rpt5a* mutants under the normal B condition (Figure 2A). However, high-B stress caused reduction in the stele width only in *rpt5a* mutants (Figure 2A). Consistently, significant reduction in the number of cell files was observed only in *rpt5a-4* treated with high-B stress (Figure 2B). These results suggested that RPT5a is involved in the maintenance of stele organization under the high-B condition but not under the normal B condition.

The number of cell files in the stele is determined by the regulation of the periclinal cell divisions of procambium cells produced around the stem cell niche (Levesque et al., 2006). It was expected that defects in the stele organization in *rpt5a* mutants under high-B stress was attributable to the disorganization of the stem cell niche where the *RPT5a* promoter was activated under high-B stress (Figure 1). Indeed, root tips of *rp5a-4* showed severe defects in alignment of cell files around the stem cell niche under the high-B condition (Figure 2B,C) (Sakamoto et al., 2018). In addition, we observed the highly disordered expression of *pWOX5::erGFP*, which is a marker for QC cells (Blilou et al., 2005), in *rpt5a* mutants treated with high-B stress, which represented the disorganization of the stem cell niche (Figure 2C). Although slightly broader expression of *pWOX5::erGFP* was observed (Figure 2C), the stele width was not affected in *rpt5a* mutants under the normal B condition (Figure 2A). Taken together, these results suggested that the function of RPT5a in the maintenance of the stem cell niche is especially crucial for organization of the RAM under high-B stress.

### RPT5a Is Crucial for Maintaining Auxin Responses and Transport in the RAM Under High-B Stress

**FIGURE 3:**
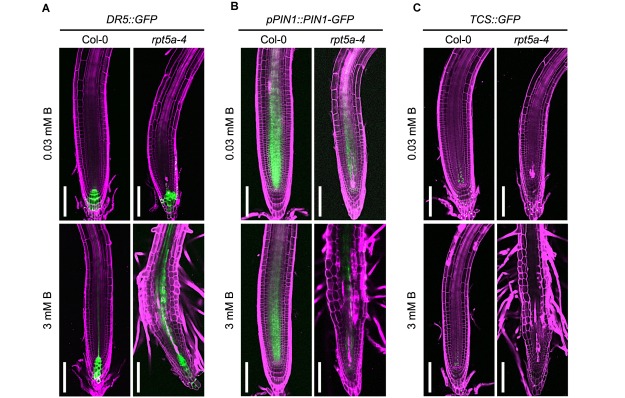
Effects of high-B stress on auxin and cytokinin signaling in the *rpt5a* mutant. **(A–C)** Expression patterns of an auxin accumulation reporter, *DR5*:GFP **(A)**, an auxin transport protein, *pPIN1*::PIN1-GFP **(B)**, and a cytokinin signaling reporter *TCS::GFP* **(C)** in Col-0 and *rpt5a-4* under the normal (0.03 mM B) and high-B (3 mM B) conditions. Scale bars, 100 μm. Magenta, PI-stained cell walls; green, GFP fluorescence.

The feedback circuit between WUSCHEL RELATED HOMEOBOX (WOX5) and an auxin response repressor, IAA17, is essential for patterning of the auxin gradient in the root tip, which is crucial for organization of the stem cell niche (Tian et al., 2014). Therefore, the dysregulation of WOX5 expression led us to speculate on the collapse of proper auxin response and distribution in the *rpt5a* mutant. To evaluate this hypothesis, we used the *DR5*::GFP reporter for auxin accumulation (Ottenschläger et al., 2003), and the *pPIN1*::*PIN1-GFP* auxin transport reporter, which acts in the stele (Benková et al., 2003). High-B treatment slightly increased *DR5::GFP* expression in the stele of the RAM in the wild type (Figure 3A). In contrast, the *rpt5a* mutant displayed highly enhanced expression of *DR5*::GFP in the stele of the RAM and differentiated regions under the high-B condition (Figure 3A). The *rpt5a-4* mutant showed reduced intensity of PIN1 expression in the stele of the RAM, under both the normal and the high-B conditions (Figure 3B and Supplementary Figure S1). Thus, we concluded that the auxin response within the RAM was altered in the *rpt5a* mutant, especially under the high-B condition.

The periclinal division of procambium cells, which determines the width of the stele, is regulated cytokinin (Ohashi-Ito et al., 2014). Reductions in the width and number of cell files in the stele in the *rpt5a-4* mutant under high-B stress (Figure 2A,B) implied the alteration of cytokinin responses around the stem cell niche in this mutant. Analysis of the cytokinin signaling reporter *TCS::GFP* (Zürcher et al., 2013) revealed that the loss of function of RPT5a resulted in the extremely low level of *TCS::GFP* expression in the stem cell niche above the QC region, irrespective of B condition (Figure 3C). These results suggest that the cytokinin response is highly repressed in the *rpt5a* mutant, but it is unlikely to be directly associated with the disorganization of the RAM in the *rpt5a* mutant under high-B stress.

### RPT5a Is Crucial for Maintaining Auxin and Cytokinin-Responsive Gene Expression Level in the Root Tip Under High-B Stress

**FIGURE 4:**
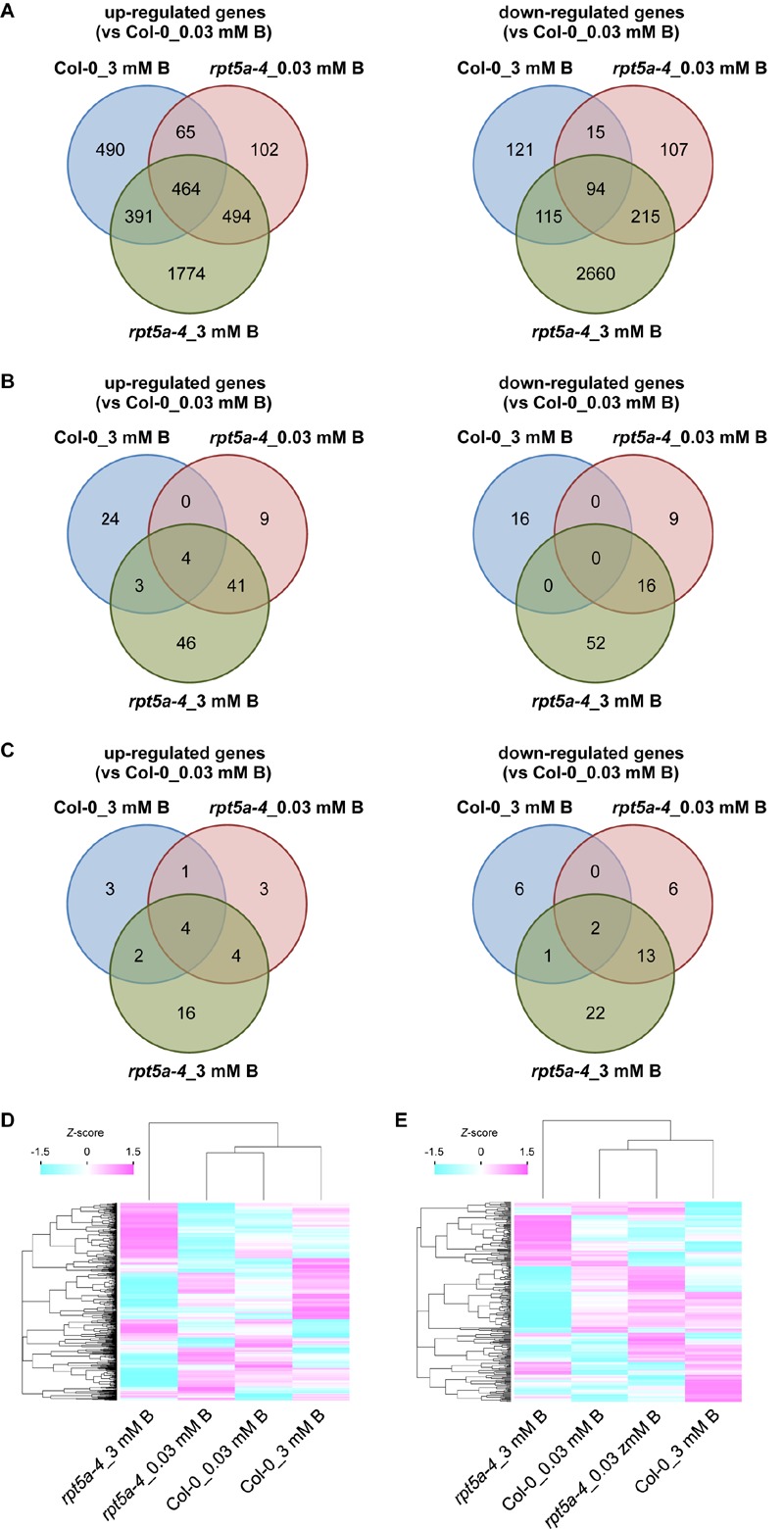
Transcriptome analysis of the *rpt5a* mutant under high-B stress. **(A)** Number of up-regulated (left) and down-regulated (right) genes in each condition. Genes that showed more than twofold change (*p* < 0.05) in expression relative to its expression level in Col-0 under the normal B condition were analyzed. **(B,C)** Number of high-B stress dependent up-regulated (left) and down-regulated (right) genes annotated with “response to auxin” (GO: 0009733) **(B)** and “response to cytokinin” (GO: 0009735) **(C)** under each condition. **(D,E)** Differences in expression levels of all genes annotated with “response to auxin” **(D)** and “response to cytokinin” **(E)** among all conditions. Heat maps show relative expression levels for the genes by *z*-scores of read counts per million mapped reads.

To further evaluate the involvement of RPT5a in the regulation of auxin and cytokinin responses under the high-B condition, we performed RNA sequencing (RNA-seq) analysis using ∼1-cm-long root tips subjected to high-B treatment for 4 days. A total of 24,758 genes were analyzed. We identified 1,410 (wild type) and 3,423 (*rpt5a-4*) up-regulated (≥2-fold, *p* < 0.05) genes and 345 (wild type) and 3,085 (*rpt5a-4*) down-regulated genes (≤0.5-fold, *p* < 0.05) in response to high-B treatment (Figure 4A). Among the genes differentially expressed in the *rpt5a-4* mutant, 2,268 up-regulated genes and 2,875 down-regulated genes were not detected in the wild type subjected to high-B stress (Figure 4A), which indicated that the loss of function of RPT5a was predominantly responsible for the substantial changes in gene expression profiles in root tips under high-B stress. The gene ontology analysis showed that genes associated with primary and secondary metabolic processes were significantly enriched in the high-B-dependent up-regulated gene sets in both the wild type and the *rpt5a-4* mutant (Supplementary Figure S2A and Supplementary File S1). In the case of the gene set down-regulated by high-B stress, genes associated with metal ion homeostasis were highly enriched in the wild type, whereas genes associated with cell wall organization were highly enriched in the *rpt5a-4* mutant (Supplementary Figure S2B and Supplementary File S1).

Next, we focused on auxin- and cytokinin-responsive genes. Among genes up- and down-regulated by high-B stress, the number of genes annotated with “response to auxin” (GO: 0009733) was greater in the *rpt5a-4* mutant (94 up-regulated, 68 down-regulated) compared with those in the wild type (31 up-regulated, 16 down-regulated) (Figure 4B). We observed that *ARF5*, a transcriptional activator for auxin-responsive genes that is expressed in the stele (Rademacher et al., 2011), was up-regulated even in the *rpt5a-4* mutant under the normal B condition and was further induced by high-B stress in both the wild type and the *rpt5a-4* mutant (Supplementary Figure S2C and Supplementary File S2). In addition, a gene set specifically up-regulated in *rpt5a-4* including *ARF11* which is also expressed in RAM including the stele (Rademacher et al., 2011) (Supplementary Figure S2C and Supplementary File S2). In contrast, a gene set specifically down-regulated in the *rpt5a-4* mutant involved auxin transport genes, such as *PIN2* and *PIN4* (Blilou et al., 2005; Grieneisen et al., 2007), and negative regulators of auxin-responsive gene expression, such as *IAA6* and *IAA7* (Tiwari et al., 2001) (Supplementary Figure S2D and Supplementary File S2). Regarding genes annotated with “response to cytokinin” (GO: 0009735), the number of high-B-dependent up- and down-regulated genes in the *rpt5a-4* mutant (26 and 38 genes, respectively) was also greater than that in the wild type (10 and 8 genes, respectively). A gene set specifically down-regulated in the *rpt5a-4* mutant included the cytokinin receptor *AHK5* (Kurepa et al., 2014), and positive and negative regulators for cytokinin responses, namely *ARR12* and *ARR8*, respectively (To and Kieber, 2008) (Supplementary Figure S2E and Supplementary File S2). Although the difference in expression was not less than 0.5-fold, the expression of *WOL/AHK4/CRE1*, a cytokinin receptor involved in stele development (Mähönen et al., 2000), was also significantly reduced in the *rpt5a-4* mutant, especially under the high-B condition (Supplementary Figure S2E). When we compared the differences in expression of whole genes annotated as auxin- and cytokinin-responsive genes, both expression profiles were drastically different, especially for *rpt5a-4* under high-B stress (Figure 4D,E). In conclusion, these data further support the hypothesis that RPT5a acts in modulating auxin response and distribution and cytokinin response, especially under the high-B condition. It should be noted that we could not completely exclude the possibility that the differences in gene expression were merely attributable to the differences in composition of cells in 1-cm-long root tips.

### Repression of TIR1/AFB-Dependent Auxin Signaling Pathway by RPT5a Is Crucial for Maintenance of RAM Morphology Under High-B Stress

**FIGURE 5:**
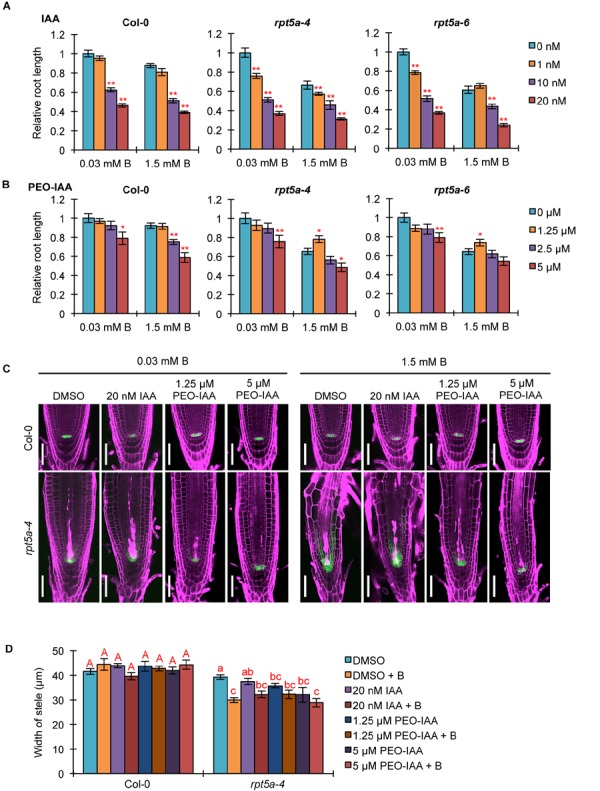
Effects of alteration in auxin signaling on the hypersensitivity of roots of the *rpt5a* mutant to high-B stress. **(A,B)** Effect of IAA **(A)** or PEO-IAA **(B)** treatments on the sensitivity of root growth in Col-0 and *rpt5a* mutants to high-B stress. The root length is represented as a ratio relative to that of untreated seedlings (defined as 1). Values are means ± SE (*n* = 15–19 for IAA, *n* = 19–24 for PEO-IAA, ^∗^*p* < 0.05, ^∗∗^*p* < 0.01, Student’s *t*-test). **(C)** Effects of IAA or PEO-IAA treatment on root apical meristem (RAM) morphology and expression patterns of *pWOX5::erGFP* in RAM under the normal (0.03 mM B) and high-B (1.5 mM B) conditions. Scale bars, 50 μm. Magenta, PI-stained cell walls; green, GFP fluorescence. **(D)** Effects of IAA or PEO-IAA treatment on the width of the stele at the border of the RAM in Col-0 and *rpt5a-4* under the normal and high-B conditions. Values are means ± SE (*n* = 10, *p* < 0.05, one-way ANOVA and Tukey’s HSD).

We investigated the association of the enhanced auxin responses with the hypersensitivity of the *rpt5a* mutant to high-B stress. First, we analyzed the effect of an exogenous synthetic auxin IAA on the sensitivity of root growth to high-B stress. Under the normal B condition, both *rpt5a-4* and *rpt5a-6* mutants displayed highly reduced root elongation, even under 1 nM IAA treatment in which the wild type grew normally (Figure 5A), indicating that the *rpt5a* mutant is more sensitive to auxin with respect to root elongation. Simultaneous treatment of IAA and 1.5 mM B reduced the apparent sensitivity of root growth to high-B stress expressed as the root elongation ratio relative to that under the normal B condition in *rpt5a* mutants (∼60–65% without IAA and 75–85% with 10 nM IAA treatment), but not in the wild type (∼82–87% in all conditions) (Figure 5A). These results suggested that B has actions similar to those of IAA in roots of the *rpt5a* mutant. Next, we investigated the involvement of the TIR1/AFB-dependent auxin signaling pathway in the sensitivity of root growth to high-B stress using PEO-IAA, an inhibitor of the TIR1/AFB-dependent auxin signaling pathway. The negative effect of PEO-IAA on root growth under the normal B condition was comparable between the wild type and the *rpt5a* mutants. However, the effect of PEO-IAA under the high-B condition differed between the wild type and the *rpt5a* mutants. In *rpt5a-4* and *rpt5a-6*, 1.25 μM PEO-IAA treatment alleviated the inhibitory effect of high-B stress on root growth (Figure 5B). In addition, the negative effect of higher concentrations of PEO-IAA on root growth was less than that in the wild type under the high-B condition (Figure 5B). The present results suggested that the enhancement in auxin responses through the TIR1/AFB-dependent auxin signaling pathway is a critical cause of the severe inhibition of root growth in the *rpt5a* mutant under high-B stress.

We observed the effect of enhanced auxin responses on RAM morphology in the *rpt5a* mutant under the high-B condition. Concurrently, the expression pattern of *pWOX5::erGFP* as a marker for maintenance of the stem cell niche was observed. Although a high concentration of IAA (20 nM) caused severe inhibition of root growth in *rpt5a* mutants, irrespective of B condition (Figure 5A), the RAM morphology and *WOX5* expression in *rpt5a-4* were similar to those observed without IAA treatment (Figure 5C). In contrast, 1.25 μM PEO-IAA treatment prevented the alteration of RAM morphology and the extent of cells expressing *pWOX5::erGFP* under high-B stress in the *rpt5a-4* mutant (Figure 5C), which is likely associated with the improved root growth (Figure 5B). Similar effects were observed in *rpt5a-4* treated with a higher concentration of PEO-IAA (5 μM), even though root growth was not recovered (Figure 5B). We should note that improvement of RAM maintenance by PEO-IAA treatment was not accompanied by increase in the width of the stele (Figure 5C), which suggested that maintenance of the stele width requires certain cellular processes subsequently for maintenance of the stem cell niche.

Analysis of *DR5::GFP* expression confirmed that IAA treatment did not further enhance the local auxin maximum in the RAM of *rpt5a-4* upon high-B stress (Supplementary Figure S3), consistent with the results observed for RAM morphology (Figure 5C). Even 1 μM PEO-IAA treatment significantly inhibited *DR5* activity (Hayashi et al., 2012). However, *DR5::GFP* expression remained high in the RAM of *rpt5a-4* under the high-B plus PEO-IAA conditions (Supplementary Figure S3). Considering that *DR5*-inducible expression depends on TIR1/AFB (De Smet et al., 2007) and PEO-IAA inhibits TIR1/AFB activity (Hayashi et al., 2012), the increased *DR5::GFP* expression in *rpt5a-4* under the high-B plus PEO-IAA conditions might be mediated by a different pathway other than TIR1/AFB auxin signaling. It is also likely that the inhibition of certain components of auxin responses is sufficient to prevent the defects in RAM maintenance.

Taken together, these results established that RPT5a is involved in the repression of the TIR1/AFB-dependent auxin signaling pathway, which is crucial for the maintenance of RAM morphology under high-B stress.

### RPT5a Is Involved in Regulation of Auxin/Cytokinin Balance in RAM Maintenance, Especially Under High-B Stress

**FIGURE 6:**
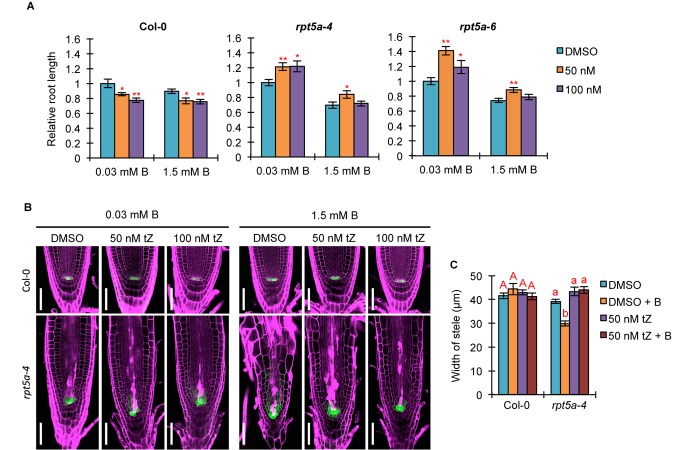
Effects of increased cytokinin treatment on the hypersensitivity of roots of the *rpt5a* mutant to high-B stress. **(A)** Effects of tZ treatment on sensitivity of root growth in Col-0 and *rpt5a* mutants to high-B stress. The root length is represented as a ratio relative to that of untreated seedlings (defined as 1). Values are means ± SE (*n* = 19–22, ^∗^*p* < 0.05, ^∗∗^*p* < 0.01, Student’s *t*-test). **(B)** Effects of tZ treatment on RAM morphology and expression patterns of *pWOX5::erGFP* in the RAM under the normal (0.03 mM B) and high-B (1.5 mM B) conditions. Scale bars, 50 μm. Magenta, PI-stained cell walls; green, GFP fluorescence. **(C)** Effects of tZ treatment on the width of the stele at the border of the RAM in Col-0 and *rpt5a-4* under the normal and high-B conditions. Values are means ± SE (*n* = 10, *p* < 0.05, one-way ANOVA and Tukey’s HSD).

Auxin and cytokinin function antagonistically in controlling cell division and differentiation for root growth (Lee et al., 2013). Given that *TCS::GFP* expression in the RAM was highly reduced in the *rpt5a* mutant, irrespective of B condition (Figure 2A), we speculated that defective cytokinin signaling causes an inclination of the auxin/cytokinin balance toward auxin, especially under the high-B condition. To evaluate this hypothesis, we analyzed the effect of treatment with the synthetic cytokinin tZ on root growth and morphology. Interestingly, we observed that 50 nM tZ improved root growth in the *rpt5a-4* and *rpt5a-6* mutants under the normal B condition, whereas root growth of the wild type was inhibited (Figure 6A). Similarly, 50 nM tZ treatment improved root growth in *rpt5a* mutants but not in the wild type (Figure 6A). These results indicated that reduced root growth in *rpt5a* mutants was attributable to reduced cytokinin signaling in the RAM. We also observed that *rpt5a* mutants displayed a reduced ratio in root elongation under the high-B plus tZ conditions (∼70–75% without tZ and ∼60–70% with tZ). Therefore, with regard to the sensitivity of root growth of *rpt5a* mutants to high-B stress, tZ alone was ineffective in negating the hypersensitivity to high-B stress of root growth. However, confocal microscopic analyses of RAM morphology indicated that cytokinin treatment conferred the *rpt5a-4* mutant with tolerance to high-B stress with regard to maintenance of the stem cell niche represented by PI staining and *pWOX5::erGFP* expression (Figure 6B) and the width of the stele (Figure 6C). Taken together, it is plausible that the reduced cytokinin response is a cause of enhanced auxin signaling leading to the defects in RAM maintenance in the *rpt5a* mutant under high-B stress. Similar to the effect of PEO-IAA treatment, the *DR5::GFP* expression level was increased in the RAM of *rpt5a-4* under the high-B plus tZ conditions (Supplementary Figure S4), which similarly implied the possibility that enhancement of certain components of auxin responses may be sufficient to cause disorganization of the RAM in the *rpt5a* mutant under high-B stress.

## Discussion

In this study, the repression of certain components of auxin responses by RPT5a was indicated to be crucial for the maintenance of RAM morphology under high-B stress. In *A. thaliana*, in addition to the majority of other RP subunits, *RPT5* is encoded by two gene copies, *RPT5a* and *RPT5b* (Book et al., 2010). Given that the *rpt5b* mutant is not hypersensitive to high-B stress (Sakamoto et al., 2018), it is considered that 26SP containing RPT5b does not function in the repression of auxin responses in roots under high-B stress.

Considering the well-known function of 26SP in degradation of AUX/IAA family proteins that inhibit auxin responses (Kong et al., 2016), it is expected that defects in 26SP activity lead to increased stabilization of such proteins and consequently further repress the auxin responses. Indeed, the protease activities of 26SP are reduced in *rpt5a* mutants, irrespective of B condition (Sakamoto et al., 2018). However, *DR5::GFP* expression and transcriptome data indicated that auxin responses were enhanced compared with those of the wild type, especially under the high-B condition (Figure 3, 4 and Supplementary Figure S2). This finding could be explained by the existence of certain factors, such as PTRE1, that enhance 26SP activities regarding the degradation of AUX/IAA family proteins independently of the TIR1/AFB pathway (Yang et al., 2016). Interestingly, consistent with this possibility, the expression of *PTRE1* was prone to be up-regulated in the *rpt5a-4* mutant (Supplementary Figure S5). Combined with the observed improvement of RAM morphology in the *rpt5a* mutant under high-B stress by PEO-IAA treatment, which stabilizes AUX/IAA proteins (Figure 5), it is considered that 26SP functions to fine-tune the homoeostasis of AUX/IAA proteins to maintain the auxin responses required for RAM maintenance at the appropriate level under the high-B condition.

In the *rpt5a* mutant, *ARF5* was highly up-regulated under high-B stress (Supplementary Figure S2), which is upstream of TMO5 and T5L1 (De Rybel et al., 2013). TMO5–LHW and T5L1–LHW regulate both the activation and inhibition of periclinal division of procambium cells in the stem cell niche in a cytokinin-dependent manner (Ohashi-Ito et al., 2014). However, *rpt5a* mutants showed reduced width and cell files of the stele, which is determined by the number of cell files in the stem cell niche (Figure 2). The phenotype of the *rpt5a* mutant may be due to the reduced cytokinin responses, which was suggested by low *TCS::GFP* expression and insensitivity to external cytokinin in terms of root growth (Figure 3, 5). We observed that *TMO5* was up-regulated and its downstream gene *AHP6*, a repressor of the cytokinin responses required for periclinal cell division, was also up-regulated, whereas the expression level of activators of cytokinin responses, *LOG3* and *LOG4*, was unchanged (Supplementary Figure S5). The present data also suggested that the activity of cytokinin-dependent periclinal cell divisions was reduced in the *rpt5a* mutant, especially under high-B stress. Promotion of cytokinin responses by treatment with tZ rescued the width of the stele in the *rpt5a* mutant under the high-B condition (Figure 5). This result suggested that 26SP containing RPT5a is involved in switching of the cytokinin response toward activation, although the molecular mechanism remains unknown. Based on this hypothesis, the reason that the width of the stele was not rescued by PEO-IAA treatment in the *rpt5a* mutant under high-B stress (Figure 5) may be naturally low cytokinin responses for stem cell division.

The formation of an auxin gradient in the RAM mediated by a series of auxin transporters is indispensable for maintenance of the stem cell niche (Scheres and Krizek, 2018). A chromatin remodeling factor, BRM, directly activates auxin efflux transporters *PIN1*, *PIN2*, *PIN3*, *PIN4*, and *PIN7*, which is required for stem cell maintenance (Yang et al., 2015). Our recent finding of increased accumulation of BRM in the *rpt5a* mutant, especially under high-B stress (Sakamoto et al., 2018), implies that auxin response is enhanced. However, this finding is partially contradictory to the present results. We observed that PIN1 expression, and *PIN2* and *PIN4* transcription are highly repressed in the *rpt5a* mutant under high-B stress (Figure 2 and Supplementary Figure S2). One possible explanation for this expression pattern of *PIN* paralogs is the reduction of production and/or maintenance of cells that have the ability to express *PIN* genes, which is attributable to the defects in stele development in *rpt5a* mutant. Alternatively, it is possible that in the context of high-B stress, BRM might not act predominantly in gene regulation. Instead, it might act in loosening of chromatin structure, which becomes a critical cause of DNA damage under high-B stress, as we previously observed (Sakamoto et al., 2018).

The highly enhanced auxin response in the *rpt5a* mutant evokes the question; why is the auxin response up-regulated? Auxin is crucial for condensation of chromatin in proliferative cells, which prevents the incidence of DNA damage and affects gene regulation associated with chromatin structure (Hasegawa et al., 2018). The *rpt5a* mutant exposed to high-B stress accumulates a high level of DNA damage in the RAM (Sakamoto et al., 2018). Therefore, the enhanced auxin response in the *rpt5a* mutant may be a defensive response that modulates chromatin integrity and gene regulation in response to the incidence of DNA damage. This may also be applicable to the wild type, as the slight stimulation of auxin signaling represented by *DR5::GFP* and *ARF5* expression was also observed in the wild type under high-B stress (Figure 2 and Supplementary Figure S2).

## Conclusion

In conclusion, the present results have established novel roles for 26SP containing RPT5a in regulation of both auxin and cytokinin responses to maintain the stem cell niche and consequent RAM morphology. In this context, 26SP containing RPT5a may regulate multiple proteins to modulate the auxin/cytokinin balance associated with the formation of proper stem cell alignment and the activity of stem cell division that enables the proper development of vascular tissues in the root under the high-B condition.

## Author Contributions

TSa and SM conceived the project and wrote the manuscript. TSa, NS, TF, and SM designed the experiments. TSa, NS, and TSu performed the experiments. TSa analyzed the data. All authors read and approved the final version of the manuscript.

## Conflict of Interest Statement

The authors declare that the research was conducted in the absence of any commercial or financial relationships that could be construed as a potential conflict of interest.

## Footnotes

1 http://rsb.info.nih.gov/ij/

2 http://geneontology.org/page/go-enrichment-analysis

## Supplementary Material

FIGURE S1 Quantitative analysis of PIN1-GFP expression. Mean intensity of PIN1-GFP signal in the RAM was measured using ImageJ software (*n* = 3, *p* < 0.05, one-way ANOVA and Tukey’s HSD).

FIGURE S2 Analysis of RNA-seq data. **(A,B)** Top 20 enriched Gene Ontology terms among up-regulated **(A)** and down-regulated **(B)** genes in Col-0 (left panel) and the *rpt5a-4* mutant (right panel) under the 3 mM B condition (*p* < 0.05, Fisher’s exact test). **(C,D)** Expression levels of selected auxin-responsive up-regulated genes **(C)** and down-regulated genes **(D)** in the RNA-seq data. **(E)** Expression levels of selected cytokinin-responsive down-regulated genes in the RNA-seq data. **(C–E)** Values are means ± SE (*n* = 4, ^∗^*p* < 0.05, ^∗∗^*p* < 0.01, Student’s *t*-test).

FIGURE S3 Effects of IAA or PEO-IAA treatment on expression patterns of *DR5::GFP* in the root apical meristem under the normal (0.03 mM B) and high-B (1.5 mM B) conditions. Five-day-old seedlings were treated with IAA or PEO-IAA for 4 days. Scale bars, 100 μm. Magenta, PI-stained cell walls; green, GFP fluorescence.

FIGURE S4 Effects of tZ treatment on expression patterns of *DR5::GFP* in the root apical meristem under the normal (0.03 mM B) and high-B (1.5 mM B) conditions. Five-day-old seedlings were treated with 50 or 100 nM tZ for 4 days. Scale bars, 100 μm. Magenta, PI-stained cell walls; green, GFP fluorescence.

FIGURE S5 Expression levels of selected genes in RNA-seq data. Values are means ± SE (*n* = 4, ^∗^*p* < 0.05, ^∗∗^*p* < 0.01; Student’s *t*-test).

FILE S1 List of significantly enriched gene ontology terms.

FILE S2 List of genes annotated with “response to auxin” and “response to cytokinin” among differentially expressed genes.
