# Supplementary material for: The 26S Proteasome Is Required for the Maintenance of Root Apical Meristem by Modulating Auxin and Cytokinin Responses Under High-Boron Stress
Source: Front Plant Sci. 2019 May 14;10:590. doi: 10.3389/fpls.2019.00590 (PMC6530338; doi:10.3389/fpls.2019.00590)
Supplement: FIGURE S1 — Quantitative analysis of PIN1-GFP expression. Mean intensity of PIN1-GFP signal in the RAM was measured using ImageJ software (n = 3, p < 0.05, one-way ANOVA and Tukey’s HSD). [file Data_Sheet_1.PDF]

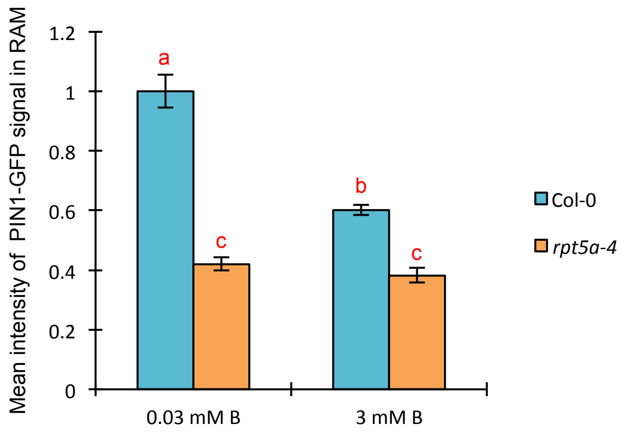

SUPPLEMENTARY FIGURE S1. Quantitative analysis of PIN1-GFP expression. Mean intensity of PIN1-GFP signal in the RAM was measured using ImageJ software ($n = 3$, $p < 0.05$, one-way ANOVA and Tukey's HSD).

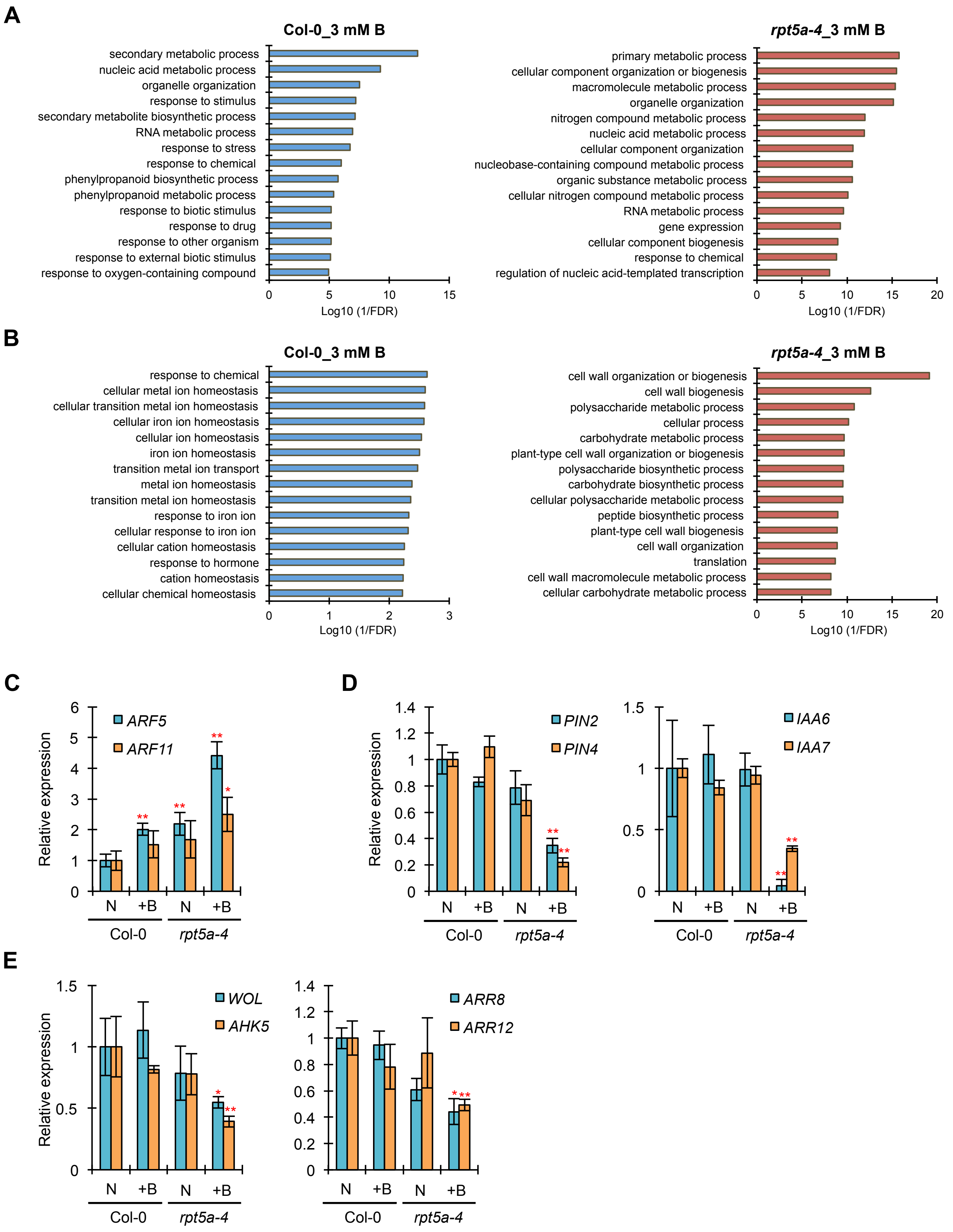

SUPPLEMENTARY FIGURE S2. Analysis of RNA-seq data. **(A, B)** Top 20 enriched Gene Ontology terms among up-regulated **(A)** and down-regulated **(B)** genes in Col-0 (left panel) and the *rpt5a-4* mutant (right panel) under the 3 mM B condition ($p < 0.05$, Fisher's exact test). **(C, D)** Expression levels of selected auxin-responsive up-regulated genes **(C)** and down-regulated genes **(D)** in the RNA-seq data. **(E)** Expression levels of selected cytokinin-responsive down-regulated genes in the RNA-seq data. **(C–E)** Values are means ± SE ($n = 4$, *$p < 0.05$, **$p < 0.01$, Student's t-test).

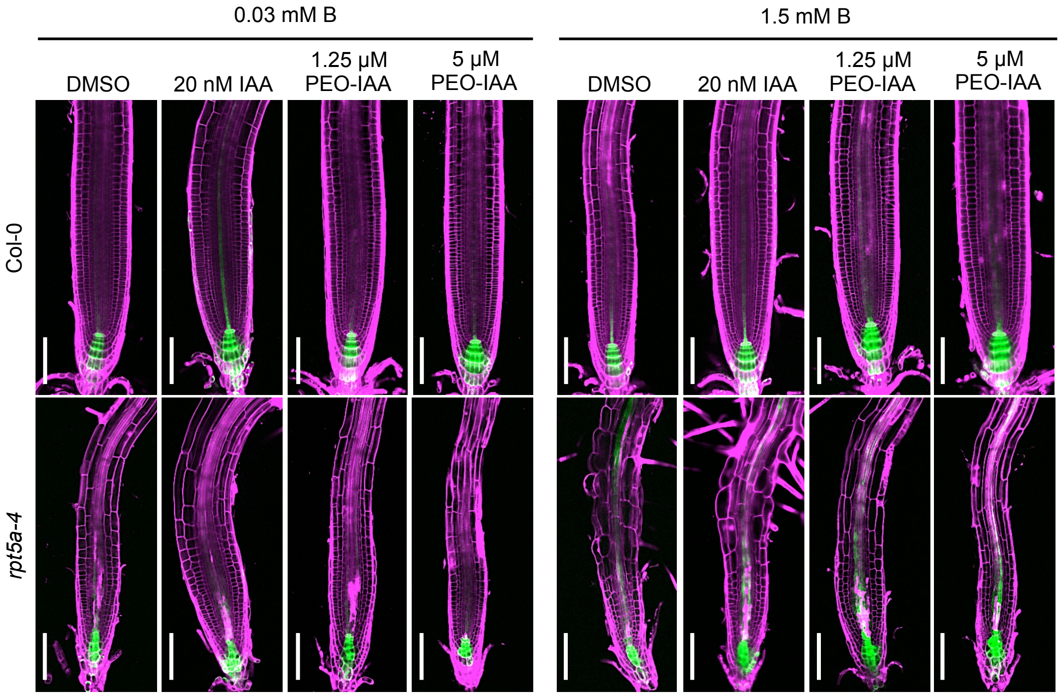

SUPPLEMENTARY FIGURE S3. Effects of IAA or PEO-IAA treatment on expression patterns of *DR5::GFP* in the root apical meristem under the normal (0.03 mM B) and high-B (1.5 mM B) conditions. Five-day-old seedlings were treated with IAA or PEO-IAA for 4 days. Scale bars, 100 µm. Magenta, PI-stained cell walls; green, GFP fluorescence.

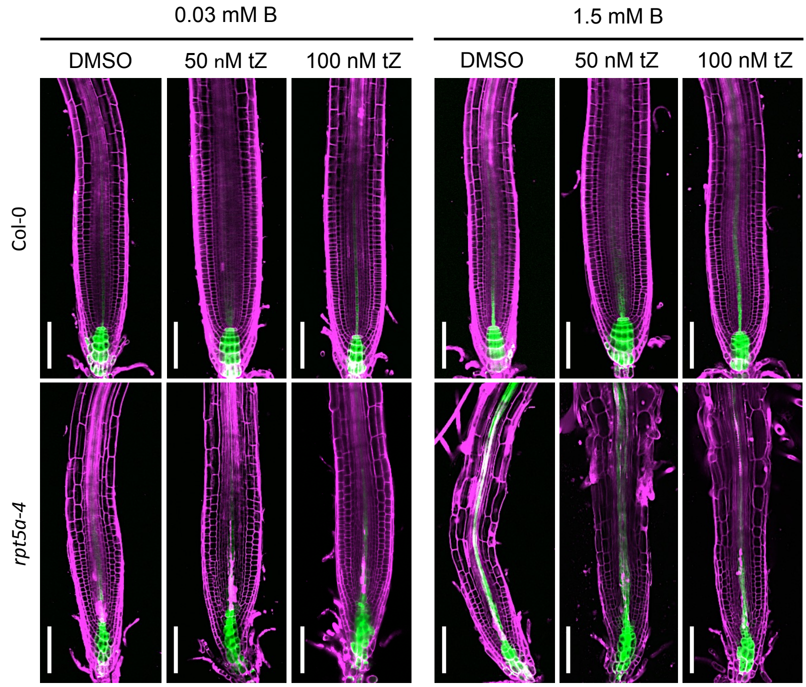

SUPPLEMENTARY FIGURE S4. Effects of tZ treatment on expression patterns of *DR5::GFP* in the root apical meristem under the normal (0.03 mM B) and high-B (1.5 mM B) conditions. Five-day-old seedlings were treated with 50 or 100 nM tZ for 4 days. Scale bars, 100 µm. Magenta, PI-stained cell walls; green, GFP fluorescence.

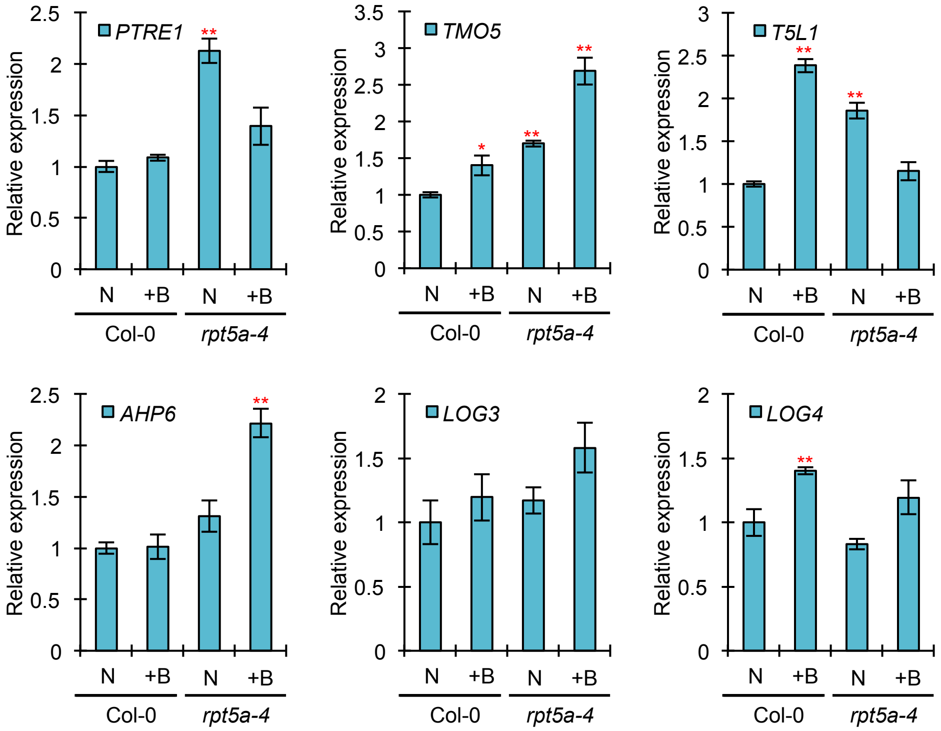

SUPPLEMENTARY FIGURE S5. Expression levels of selected genes in RNA-seq data. Values are means ± SE (n = 4, *p < 0.05, **p < 0.01; Student's t-test).
